# Differential gene expression of 3D primary human airway cultures exposed to cigarette smoke and electronic nicotine delivery system (ENDS) preparations

**DOI:** 10.1186/s12920-022-01215-x

**Published:** 2022-04-03

**Authors:** Rachael E. Rayner, Patrudu Makena, Gang Liu, G. L. Prasad, Estelle Cormet-Boyaka

**Affiliations:** 1 Department of Veterinary Biosciences, The Ohio State University, 1925 Coffey Road, Columbus, OH 43210 USA; 2 RAI Services Company, Winston-Salem, NC USA; 3 Prasad Scientific Consulting LLC, Lewisville, NC USA

**Keywords:** Primary Normal Human Bronchial Epithelial (NHBE) cells, Tobacco, Electronic Nicotine Delivery Systems (ENDS), Transcriptomics

## Abstract

**Background:**

Acute exposure to cigarette smoke alters gene expression in several biological pathways such as apoptosis, immune response, tumorigenesis and stress response, among others. However, the effects of electronic nicotine delivery systems (ENDS) on early changes in gene expression is relatively unknown. The objective of this study was to evaluate the early toxicogenomic changes using a fully-differentiated primary normal human bronchial epithelial (NHBE) culture model after an acute exposure to cigarette and ENDS preparations.

**Results:**

RNA sequencing and pathway enrichment analysis identified time and dose dependent changes in gene expression and several canonical pathways when exposed to cigarette preparations compared to vehicle control, including oxidative stress, xenobiotic metabolism, SPINK1 general cancer pathways and mucociliary clearance. No changes were observed with ENDS preparations containing up to 28 µg/mL nicotine. Full model hierarchical clustering revealed that ENDS preparations were similar to vehicle control.

**Conclusion:**

This study revealed that while an acute exposure to cigarette preparations significantly and differentially regulated many genes and canonical pathways, ENDS preparations containing the same concentration of nicotine had very little effect on gene expression in fully-differentiated primary NHBE cultures.

**Supplementary Information:**

The online version contains supplementary material available at 10.1186/s12920-022-01215-x.

## Background

Long-term cigarette smoking impacts lung biology, including induction of inflammation, altered microbial defense, and compromised immune surveillance. Several studies have demonstrated that cigarette smoke alters gene expression in different tissues including bronchial airway epithelia [1, 2], nasal epithelia [3], whole blood [4, 5] and peripheral blood mononuclear cells [6, 7]. Altered gene expression is associated with different biological processes such as xenobiotic metabolism, apoptosis, immune response, tumorigenesis and stress response, among others. Sustained perturbations in gene expression in key biological processes could drive/contribute to the development of smoking-related diseases such as lung cancer, cardiovascular disease and chronic obstructive pulmonary disease (COPD) [8–13].

Electronic nicotine delivery systems (ENDS) are battery operated devices designed to deliver nicotine without combustion [14]. ENDS products in the current marketplace are a highly heterogeneous product category, with several types of devices, a wide range of formulations of e-liquids and variable use settings (voltage, heating temperature, etc.). The aerosol generated by ENDS is less complex than that produced from combustible cigarettes. Cigarette smoke, which is generated by combustion, contains over 7000 chemicals that include several carcinogens, as well as harmful and potentially harmful constituents (HPHCs) [15]. In contrast, e-liquids generally consist of 4 main ingredients (vegetable glycerin, propylene glycol, nicotine and flavorings). However, the composition of ENDS aerosol varies depending on the flavorings and the device use conditions. For example, several investigators reported that ENDS aerosols contain far less quantities of volatile carbonyls, tobacco specific nitrosamines or polycyclic aromatic hydrocarbons, compared to cigarette smoke [16]. However, some investigators also have reported higher levels of some HPHCs in certain flavors and/or when the devices were operated under sub-ohm conditions [16].

Consistent with the chemistry of aerosols, ENDS users exhibit significantly lower biomarkers of exposure as shown in product-specific studies [17, 18] or population surveys [19]. Clinical findings of ENDS products have indicated that ENDS users have fewer or distinctly different gene expression profiles compared to cigarette smokers [18, 20, 21]. However, some studies report that former smokers who switched to ENDS vaping express shared, as well as distinct, patterns of gene expression when compared to cigarette smoking [20]. In addition, ENDS use has also shown impact on virus responses, including decreased gene expression [22].

As alluded to above, ENDS are diverse products, and the toxicogenomic effects of ENDS are still being understood. For rapid evaluation of ENDS effects, suitable in vitro systems would be necessary. The lung is a complex organ that consists of several highly differentiated cell types (e.g. ciliated cells and goblet cells), which facilitates mucociliary clearance and serves as the first-line of defense against pathogens and pollution. Therefore, in vitro models that assess lung function should ideally consist of differentiated cells that capture the functions of airway cell types. Existing monolayer cell culture models, although useful, are not necessarily differentiated; hence they may not recapitulate the functions of lung cell types. The differences in cell culture conditions (monolayer vs organoids), and differentiation status (undifferentiated vs differentiated cells) hence can yield different results.

Given the general complexity of culture conditions, there have been some studies investigating the effects of ENDS exposure in 3D differentiated airway cultures [23–25], which are more physiologically relevant than monolayer cultures. From the few 3D organoid studies, ENDS products were reported to alter significantly fewer or distinctly different gene expression profiles compared to cigarette smoke in bronchial and nasal cell cultures, similar to that reported in clinical studies [23–25]. To our knowledge, only one study to date used differentiated primary normal human bronchial epithelial (NHBE) cells at air–liquid interface (ALI), and reported differential gene expressions after ENDS exposure compared to cigarette smoke [23]. Other studies investigating the effect of ENDS/vaping on gene expression in differentiated NHBE cultures either investigated specific ingredients/flavorings [26] or used cell-lines at ALI such as BEAS-2B cell-line [27]. MucilAir™ (Epithelix) primary nasal cells have been directly exposed to ENDS aerosol and 3R4F Kentucky reference cigarette smoke, revealing that ENDS aerosol had reduced effect on gene expression [24, 25]. However, other studies have reported extensive reprogramming of gene expression, suggesting that ENDS use is associated with inflammatory responses [28]. Further investigation into the effects of cigarette and ENDS products on gene expression in fully-differentiated primary NHBE cultures are therefore required.

To address some of the challenges associated with assessing lung function, we have demonstrated the applicability of 3D lung culture models in evaluating the acute and longer-term effects of exposure to cigarette smoke and ENDS aerosol [29, 30]. Fully-differentiated primary NHBE cultures can be used to study the effect of cigarette smoke and ENDS preparations on airway mucociliary function, ion channel function, and physiological changes to the epithelium [29]. In this manuscript, to better understand the acute effects (early changes) of ENDS exposure, we utilized a transcriptomic approach with the fully-differentiated primary NHBE culture model and compared them with the profiles derived from cigarette smoke exposure. These data further support and build on our previous findings that cigarette smoke preparations, not ENDS, alter/disrupt targeted ion channel and mucociliary function, and perturb pathways related to xenobiotic and oxidative stress metabolisms.

## Results

### Exposure system on fully-differentiated primary NHBE cultures

In an effort to understand how exposures to cigarette or ENDS preparations impact gene expression in fully-differentiated primary NHBE cultures, we first examined whether treatment variables (treatment, concentration, time, donors) affected RNA-seq results (see Methods). Primary NHBE cultures from 4 donors (Additional file 2: Table S1) were fully-differentiated after 4 weeks at ALI, as determined by optimal trans-epithelial electrical resistance (TEER), and presence of ciliated and mucus producing cells [31]. Based on previously published data using lactate dehydrogenase (LDH) release as an indication of cytotoxicity [29], non-cytotoxic doses of whole-smoke conditioned medium (WS-CM) from cigarette preparations, and aerosol conditioned medium (ACM) from ENDS preparations were chosen. Low, medium, and high doses of equivalent-nicotine units (Eq-Nic.) were designated for WS-CM (3.6, 7.0 and 10.0 µg/mL Eq-Nic.) and for ACM (7.0, 14.0 and 28.0 µg/mL Eq-Nic.). Higher doses of ACM were used because no cytotoxicity was observed even at 28 µg/ml Eq-Nic units. [29].

### Next generation sequencing (NGS) analysis

NGS analysis yielded a dataset of 60,564 gene identifiers that were mapped to the Ingenuity Knowledge Base. Any duplicate identifiers were resolved to their respective genes, resulting in a total of 58,457 genes for the analysis. Samples collected at time 0 h were used for normalization.

Using unsupervised clustering identified treatment period (vehicle 0 h vs. 4 h vs. 24 h) had a strong effect on the gene expression since all the samples were primarily grouped based on exposure time (data not shown). Despite the effect of time, treatments (WS-CM and ACM) exerted an effect on gene expression in this in vitro model system. Therefore, the statistical modeling focused on different treatments/dosages within each time point to identify the differentially expressed genes (DEGs).

### WS-CM treatment exerts stronger effect on gene expression, whereas ACM elicited minimal response

WS-CM exerted marked effects on gene expression in a dose- and time-dependent fashion. Compared to vehicle control, treatment with WS-CM at low, medium and high doses of Eq-Nic. units altered expression of 6, 76, and 153 genes, respectively, after 4 h exposure using a criteria cut-off of log2 fold change > 2 or < − 2, and adjusted p-value < 0.05 (Table 1). The number of significantly regulated genes increased after a longer exposure (24 h) of WS-CM, with the low, medium and high doses of WS-CM producing 189, 281 and 388 differentially regulated genes, respectively (Table 1).

**Table 1:** Number of DEGs significantly altered by WS-CM or ACM compared to vehicle

Exposure time	WS-CM			ACM		
	Low 3.5 µg/mL	Medium 7 µg/mL	High 10 µg/mL	Low 7 µg/mL	Medium 14 µg/mL	High 28 µg/mL
4 h	6	76	153	0	0	0
24 h	189	281	388	0	0	0

In contrast, we did not observe dosage or time effect on gene expression upon treatment with ACM, per the criteria defined for statistical analysis (Table 1). Treatment with ACM resulted in no significant gene expression changes at the 3 doses tested (7.0, 14.0, and 28.0 μg/mL Eq-Nic.), which are similar or higher than the corresponding doses used for WS-CM treatment. However, when the log2 fold change cut off was lowered to > 1.5 or < -1.5 and adjusted p ≤ 0.05, the highest dose of ACM (28 µg/mL Eq-Nic.) at 4 h revealed differential expression of 8 genes (Additional file 2: Table S2). Nevertheless, even under the reduced threshold conditions, no DEGs were detected at 24 h of treatment. Furthermore, only with a consideration of a p-value ≤ 0.05, and no fold-change criteria applied, ACM treatment at 24 h did not result in the detection of any DEGs.

**Fig. 1:**
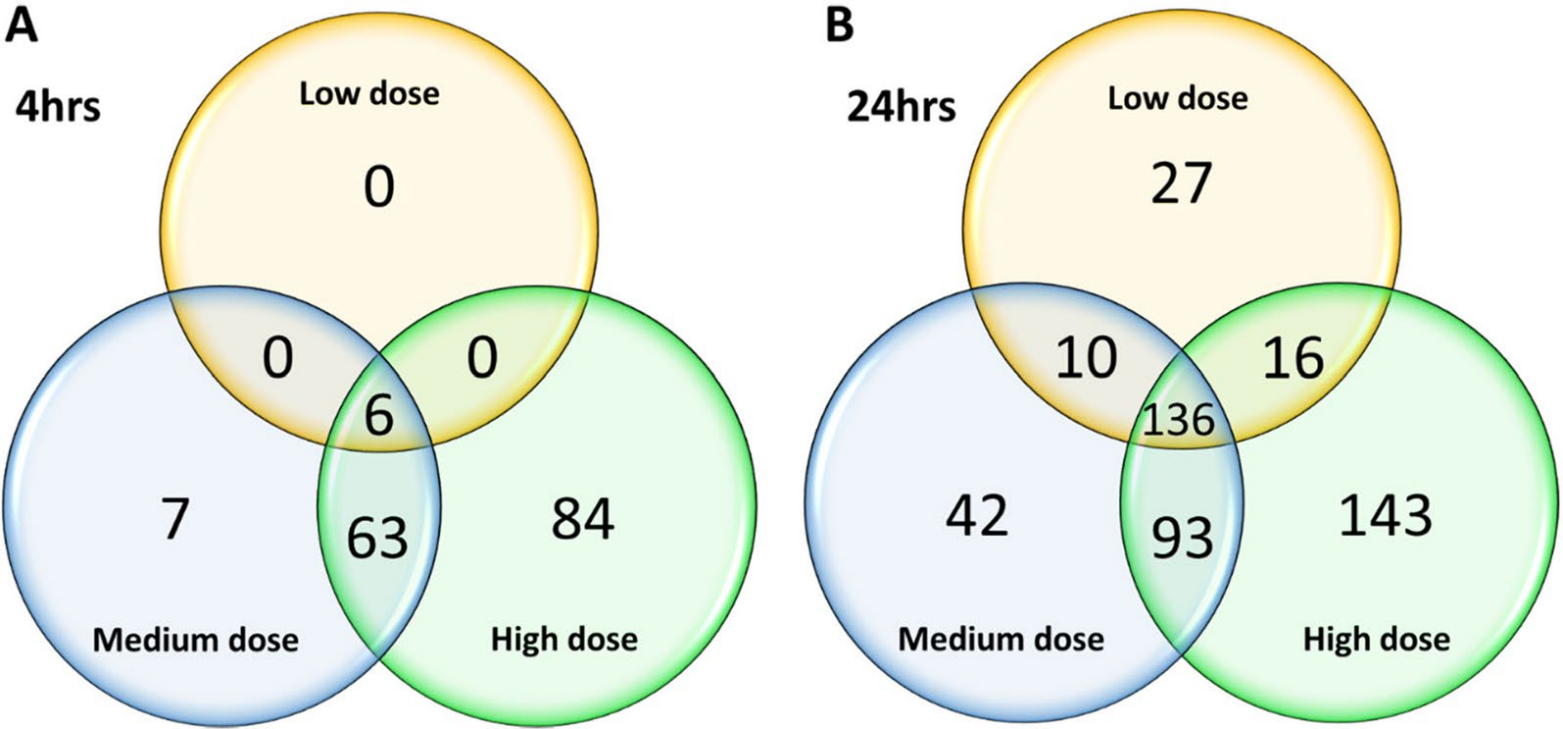
Total DEGs after exposure of fully-differentiated primary NHBE to WS-CM for 4 and 24 h. Venn diagrams showing genes differentially expressed after exposure to various doses (low, medium, and high) of WS-CM at 4 h **A**, and 24 h **B** time-points. DEGs were identified with a log2 fold change > 2 or < − 2, and adjusted p-value ≤ 0.05

Comparing across the three different doses of WS-CM, there were a few common genes at 4 h (6 genes) exposure, and 24 h (136 genes) (Fig. 1). The six differentially expressed genes (*AHRR-196, AHRR-438, ALDH1A3, LPAR6, TRBC1 and TRBC2*) after 4 h of low dose WS-CM exposure were also found to be altered at medium and high doses (Additional file 1). Only *AHRR-196, ALDH1A3* and *LPAR6* were also sustained after 24 h exposure (Additional file 1). If we only compared the medium and high doses at 4 h WS-CM exposure, 69 genes were common. When we compared the two different time-points, 4 h and 24 h WS-CM exposure, 4 and 24 genes were regulated by the medium and high dose at both time-points, respectively (Additional file 1). A set of 21 DEGs were identified in all doses and across both time-points except for low dose at 4 h. Amongst these 21 DEGs, several were increasingly upregulated with higher dose and longer exposure time (e.g. *HMOX1, CYP1A1, CYP1B1, CYP1B1-AS1*), whilst some others did not vary in expression between the two exposure times (e.g. *TIPARP, C5AR2, HILPDA, EGLN3*). A couple of genes had decreased regulation at 24 h compared to 4 h, including downregulation of *ANGPTL4* and *PGF*, suggesting that these DEGs were more strongly regulated in a shorter time period (4 h) which has begun to return to base levels by 24 h*.*

**Fig. 2:**
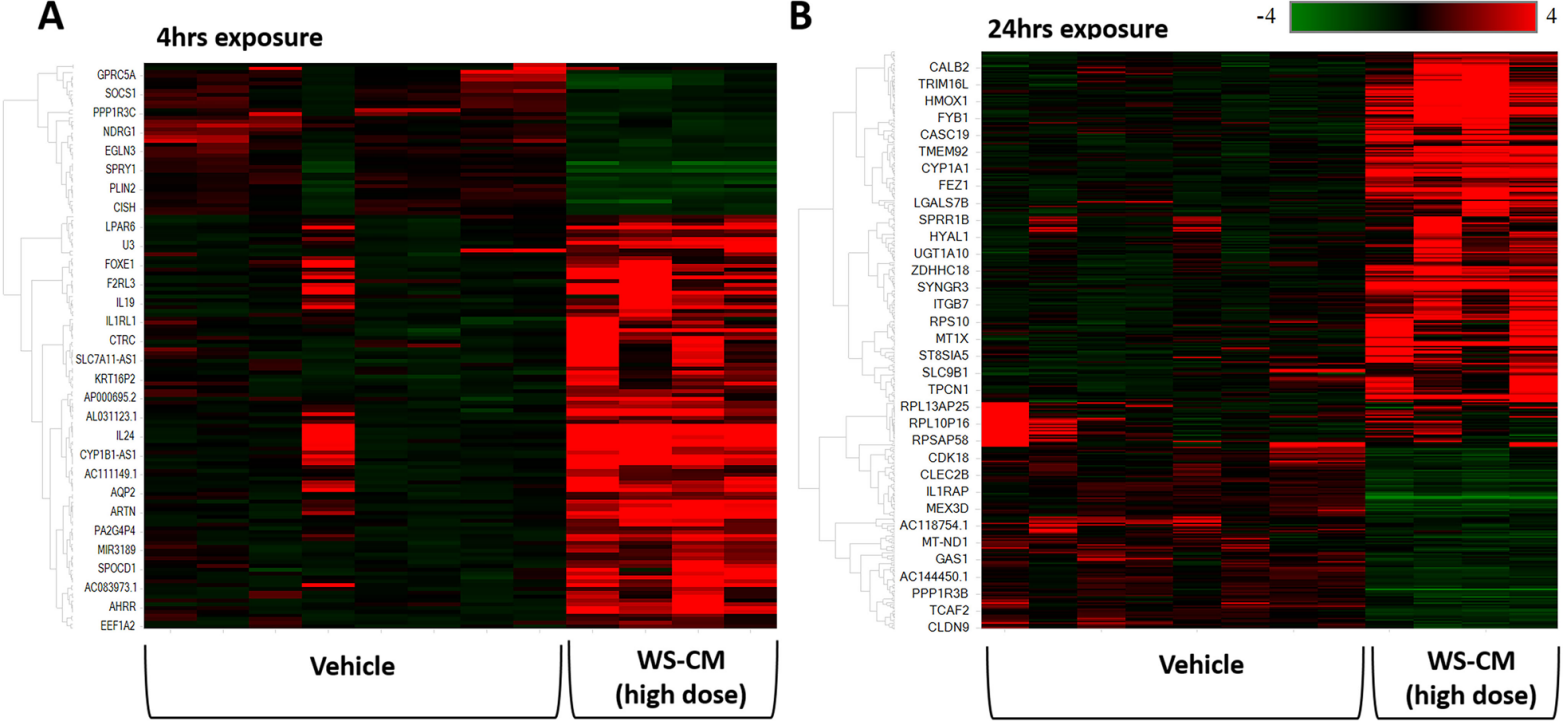
Hierarchical clustering of all DEGs which were significantly different between vehicle and high dose WS-CM. All the significant DEGs (log2 fold change > 2 or < -2, and adjusted p-value < 0.05) at 4 h (153 genes) and 24 h (388 genes), as listed in Table 1, were used for hierarchical clustering. In the heatmap, the rows represent expression values for genes, while the columns represent each sample. Low expression is denoted by green and high expression indicated by red. Compared to vehicle control, effect of WS-CM was observed after **A** 4 h exposure, and **B** 24 h exposure. N = 4 donors for each treatment; vehicle controls were performed in duplicates

Hierarchical clustering was performed to examine if DEGs would classify samples into vehicle and WS-CM-treated groups. The clustering analyses of DEGs (statistically significant p < 0.05 and ± 2 log2 fold change) allowed a clear separation of vehicle and WS-CM (Fig. 2).

**Fig. 3:**
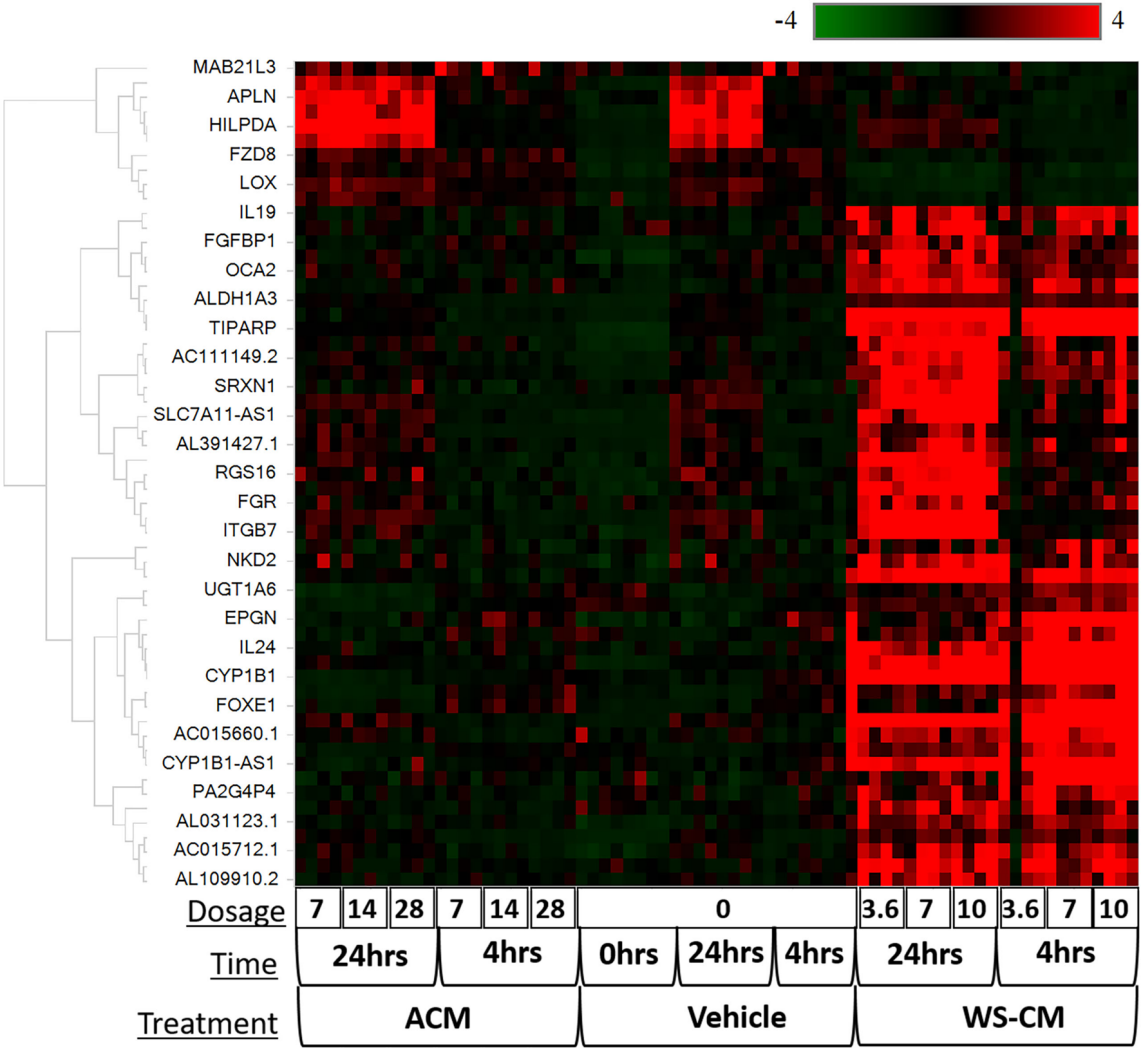
Full model hierarchical clustering of all DEGs which were significantly different between WS-CM, ACM and vehicle. In the heatmap expression values for genes are represented as rows, while the samples are shown as columns. The scale (color scheme) goes from − 4 (green for genes decreased) to 0 (black) and + 4 (red for genes increased) representing log2 FC of the genes in the different conditions. Dosage indicates nicotine concentration in the different preparations and is given in µg/mL Eq-Nic. units. DEGs identified using cut-off criteria of log2 fold change > 2 or < − 2, and adjusted p-value < 0.05

Since most of the doses of ACM tested were higher than used with WS-CM, we compared the effects of WS-CM and ACM at a common dose of 7.0 μg/ml Eq-Nic. At this dose, several genes were significantly altered by WS-CM, but not ACM at 4 h or 24 h (Fig. 3, Additional file 2: Figs. S2B and S2C). WS-CM treatment resulted in 146 DEGs, and in 303 DEGs at 4 h and 24 h, respectively, relative to ACM treatment (Additional file 2: Fig. S2A). A second method of comparison was used to determine the effect of WS-CM compared to ACM using hierarchical clustering (Additional file 2: Fig. S2B). We then attempted to identify the overall effect of treatment (WS-CM vs ACM) with time effect removed. When running full model gene expression differences between WS-CM and ACM, we identified 59 DEGs (adjusted p-value < 1E-5) that could be used as marker genes to differentiate between groups treated with WS-CM or ACM (Additional file 2: Table S3). Hierarchical clustering revealed that while all ACM-treated samples displayed a pattern similar to the control samples, the WS-CM treated samples were distinct and co-clustered (Fig. 3).

### Top DEGs identified with WS-CM exposure include those associated with phase I and II enzymes and oxidative stress

**Fig. 4:**
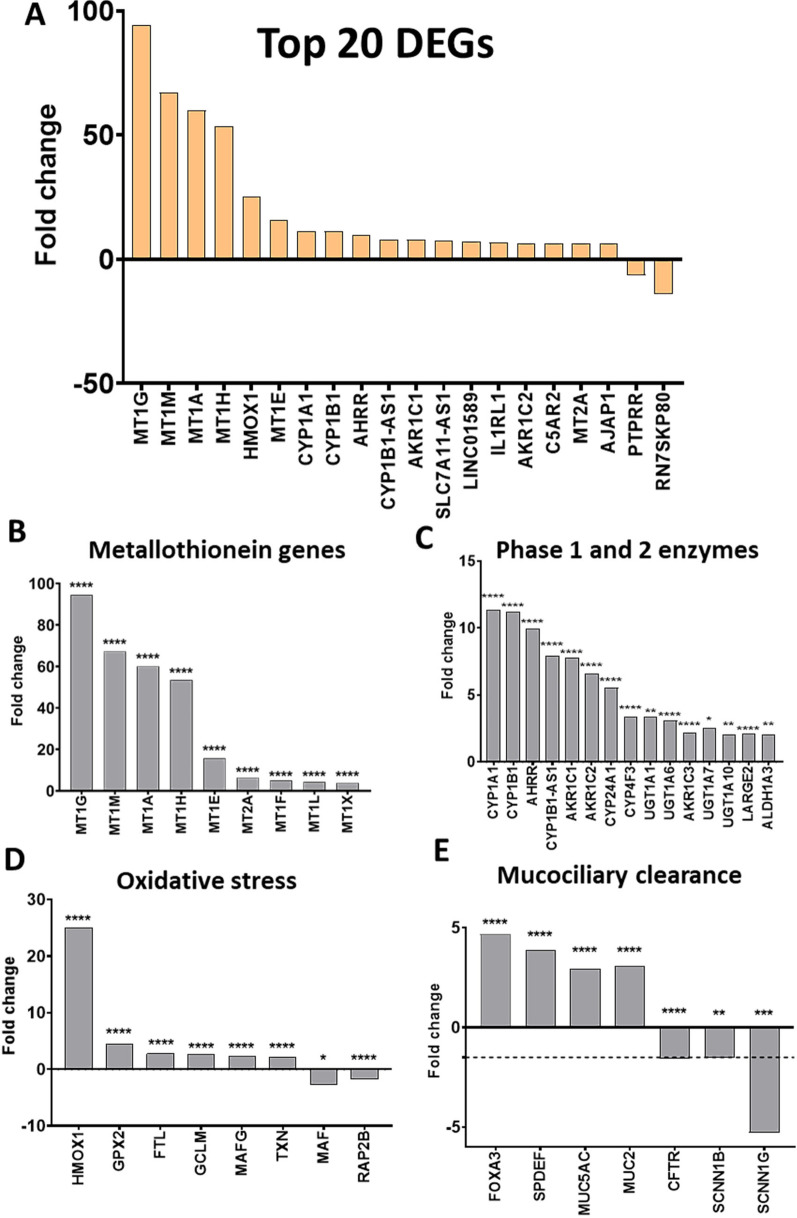
Top DEGs after exposure to high dose of WS-CM. Primary NHBE cultures were exposed to a high dose of WS-CM (10 µg/mL Eq-Nic.) for 24 h. **A** Top 20 DEGs after exposure to WS-CM. Several cellular processes were of interest including DEGs associated to metallothionein metabolism (**B**), Phase I and II enzymes xenobiotic metabolism (**C**), oxidative stress (**D**) and mucociliary clearance (**E**). Dotted line identifies the -1.5 log2 fold change cut-off, to help distinguish *CFTR* and *SCCN1B* log2 fold change which fell below -2 but was just above -1.5 (**E**). Significant DEGs were considered with log2 fold change > 2 or < -2 and adjusted p-value ≤ 0.05. *p ≤ 0.05, **p ≤ 0.01, ***p ≤ 0.001, ****p ≤ 0.0001

Treatment with WS-CM resulted in significant changes in gene expression at 4 h and 24 h, as previously shown in Table 1. The top 20 DEGs resulting from high dose treatment are shown in Fig. 4A. Metallothionein genes were most prominently induced by WS-CM after 24 h (Fig. 4B). Several xenobiotic metabolizing genes, including those responsible for the expression of phase I and phase II enzymes were also prominently induced by WS-CM. The cytochrome P450 genes, *CYP1A1*, *CYP1B1*, and *AHRR* (aryl hydrocarbon receptor repressor) genes were among those significantly upregulated by WS-CM (Fig. 4C). Genes involved in the regulation of oxidative stress such as *HMOX1* (hemoxygenase 1), *GPX* (glutathione peroxidase) and *TXN* (thioredoxin) were also induced by WS-CM treatment (Fig. 4D). Significant upregulation of *HMOX1* was observed at both 4 h and 24 h exposure to WS-CM (Additional file 2: Tables S4 and S5). Furthermore, genes involved in mucociliary function including ion channels responsible for fluid homeostasis and genes regulating differentiation of goblet cells and mucin production were also differentially regulated by WS-CM at 24 h (Fig. 4E). While genes associated with goblet cell hyperplasia and mucus secretion were up-regulated (*FOXA3, SPDEF, MUC5AC* and *MUC2*), genes encoding ion channels were down-regulated. Thus, *SCNN1G,* which encodes for the gamma subunit of the epithelial sodium channel (ENaC), was significantly down-regulated. If the log2 fold change was relaxed to ± 1.5, the beta subunit of ENaC, *SCNN1B,* was also significantly down-regulated. Another ion channel, cystic fibrosis transmembrane conductance regulator (*CFTR*) gene was significantly downregulated with cut-off log2 fold change ± 1.5. The changes to the genes associated with mucociliary function were not observed at 4 h exposure to WS-CM preparations.

### Top canonical pathways affected by WS-CM

We wanted to identify significantly associated canonical pathways, predicted upstream regulators and the top predicted diseases, and functions associated with each dataset. We used Ingenuity Pathway Analysis (IPA) to identify the differential expression analysis (see Methods for details). For the comparison analyses, the core analyses were collated and common threshold cut-offs of adjusted p-value < 0.05 and a log2 fold change threshold ± 1.5 were used to identify significant differentially expressed genes in each individual analysis. When visualizing the comparison analysis results, a Benjamini–Hochberg adjusted p-value < 0.05 and a Z-score of >  ± 2 was used to determine significance.

**Fig. 5:**
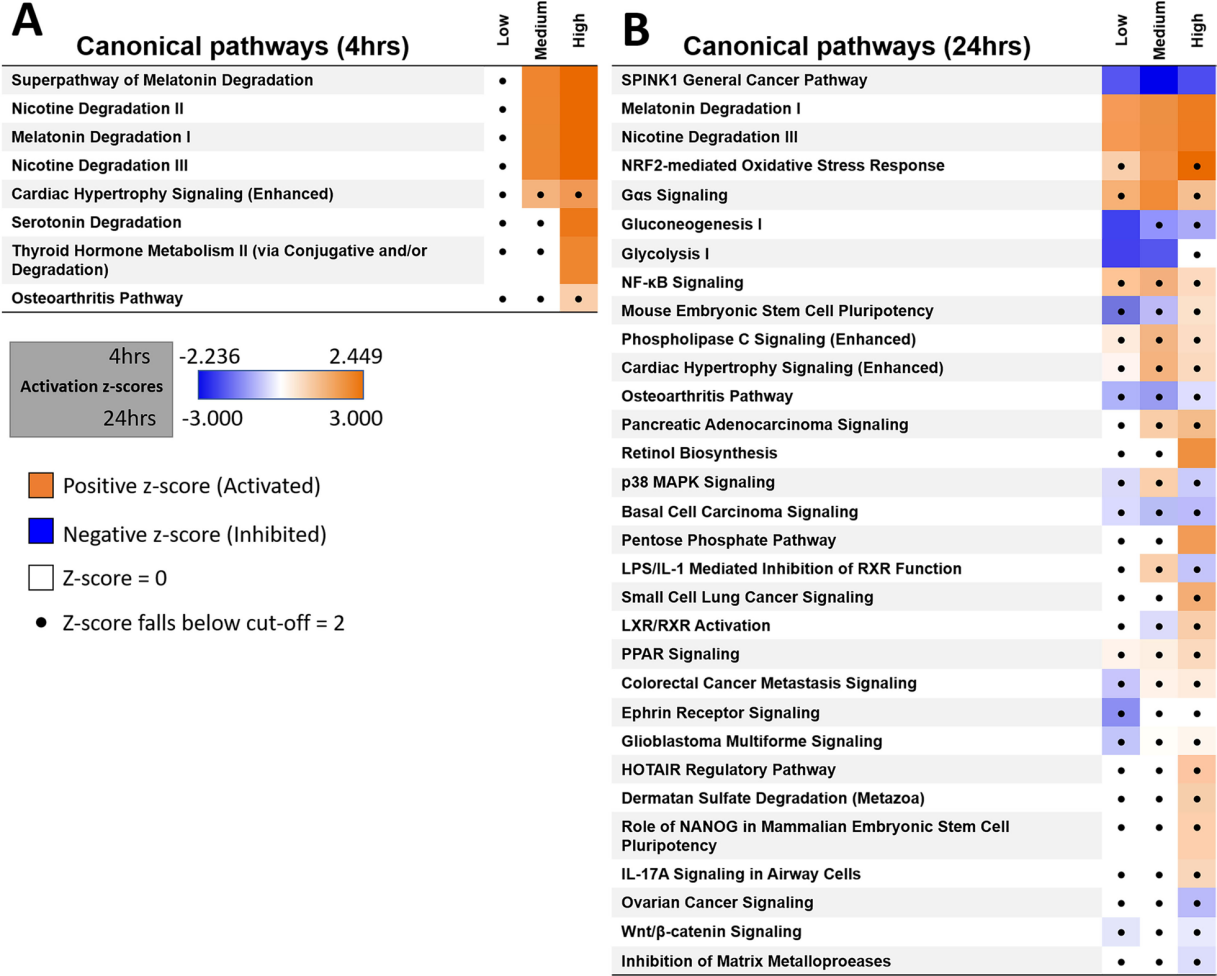
Significantly regulated canonical pathways from WS-CM dosage compared to vehicle control. Differential canonical pathways using IPA after exposure of primary NHBE cultures to low (3.6 µg/mL Eq-Nic.), medium (7.0 µg/mL Eq-Nic.) and high (10.0 µg/mL Eq-Nic.) doses of WS-CM for **A** 4 or **B** 24 h. The values in the heatmap are the z-scores of each entity in each analysis, where orange coloring indicates a predicted activation, and blue a predicted inhibition of the entity. Dots represent entities that have insignificant z-scores (defined as < 2)

The top canonical pathways identified with WS-CM (medium and high doses) compared to vehicle control after 4 h in this comparison analysis included two pathways regulating aspects of Melatonin and Nicotine Degradation (Nicotine Degradation II and Nicotine Degradation III) (Fig. 5A). Several Phase I and II enzymes are upregulated in these pathways after 4 h, including *CYP1A1, CYP1B1, UTG1A6* and *UGT1A7* (Additional file 2: Table S4)*.* A xenobiotic metabolism pathway was identified at both 4 h and 24 h, however there were not enough significantly regulated genes to determine whether this pathway was activated or inhibited.

After 24 h treatment of WS-CM, the top canonical pathways identified in this analysis included the SPINK1 general cancer pathway, nicotine degradation III, melatonin degradation and NRF-2 oxidative stress response (Fig. 5B). SPINK1 cancer pathway was predicted to be inhibited in all 3 comparisons of WS-CM *vs* vehicle control at various dosages (Fig. 5B). Several metallothionein genes, including *MT1E, MT1G* and *MT1M* were upregulated in the SPINK1 general cancer pathway (Additional file 2: Table S5). Other cancer related pathways that were altered included inhibition of gluconeogenesis I, glycolysis I, basal cell carcinoma signaling and small cell lung cancer signaling (activated) (Fig. 5B). The NRF2-mediated oxidative stress pathway was activated in all doses of WS-CM (Fig. 5B). Several oxidative stress related genes were up-regulated, including *HMOX1, GPX2, GCLM* and *TXN*, as well as down-regulated genes including *MAF* and *RAP2B* (Additional file 2: Table S5). Similar to what was observed at 4 h, nicotine and melatonin degradation pathways were activated after 24 h exposure, with several Phase I and II enzymes upregulated including *CYP1A1, CYP1B1,* and *UGT1A6* (Additional file 2: Table S5).

## Discussion

The objective of this study was to evaluate the early toxicogenomic changes after an acute exposure (4 and 24 h) of cigarette smoke preparations (WS-CM) and ENDS preparations (ACM) on fully differentiated primary human bronchial cultures. There are two major findings to be taken from this study. Firstly, extensive gene expression changes were detected due to WS-CM exposure in a time- and dose-dependent manner. ENDS preparations (ACM), on the other hand, exerted minimal effect, and the changes in gene expression resembled that of the vehicle control. Secondly, several cellular processes were differentially regulated by WS-CM including general cancer pathways and xenobiotic metabolism, oxidative stress and genes associated with mucociliary function. In contrast, no significant changes in cellular pathways were detected with ACM treatment.

As reported in this study, an acute exposure of WS-CM preparations exerted more pronounced transcriptomic changes in fully differentiated primary NHBE cultures compared to ACM. Even if the log2 fold change cut-off was lowered to ± 1.5 in this current study, only eight DEGs were identified at only the highest dose of ACM after 4 h exposure, but were not persistently seen after 24 h exposure. Other studies have demonstrated that ENDS aerosol induces less pronounced gene expression changes than cigarette smoke in primary NHBE cells; genes that were regulated by ENDS aerosol were related to oxidative stress, xenobiotic metabolism, and genes involved in cilia assembly and movement [23, 24, 32]. Two main factors may play a role in the differential effects observed between WS-CM and ACM: the chemical composition present in the preparations, and/or the formation of chemicals from the combustion of tobacco. It has been shown that heating tobacco products (HTPs), which rely on heating tobacco rather than combusting tobacco, have reduced impact on gene expression compared to 3R4F reference cigarette smoke [33].

Few studies have used fully-differentiated primary NHBE cultures for investigation of transcriptomic changes as a result of cigarette or ENDS exposure. We have expanded the use of in vitro airway models by investigating the global transcriptomic changes after an acute exposure to WS-CM and ACM preparations. Shen et al*.* [23] observed expression changes in *CYP1A1* and *HMOX1* from direct cigarette exposure (1R5F cigarette smoke) in fully-differentiated primary NHBE, similar to what we also observed in this study. Furthermore, other studies have reported regulation of genes and/or proteins by cigarette smoke in in vitro models, including metallothionein (MT1G, MT1M, MT1A) [34], CYP1B1 [35], AHRR [36], GPX2, CFTR [29, 37, 38], FOXA3, SPDEF [39, 40] and MUC5AC [29, 40]. On the other hand, expression from ENDS aerosol was distinctly different from cigarette smoke, with phospholipid and fatty acid triacylglycerol metabolism pathways significantly regulated [23]. Another in vitro study of e-cigarette liquids and aerosol reported induction of GCLM, GCLC, GPX2, NQO1 and HMOX1 [41]. Comparisons to these studies are difficult since we exposed the cells with ENDS preparation (aerosol bubbled through media) rather than aerosol or e-cigarette liquid. However, a couple of in vitro studies with e-cigarette aerosols have reported lower cytotoxicity and minimal changes in the mRNA, microRNA and protein markers [42, 43], supporting our findings with ENDS preparations. Using a less stringent cut-off of log2 FC ± 1.5, we observed a couple of nuclear proteins (Hes Related Family BHLH Transcription Factor with YRPW Motif 1 [*HEY1*] and Serum Response Factor [*SRF])* with ACM (28 µg/mL Eq-Nic.) at 4 h. The other genes appear to code for proteins related to cellular proliferation and structure, including Inertermediate Filament Family Orphan 2 (IFFO2), Cellular Communication Network Factor 2 (CCN2), FosB Proto-oncogene, AP-1 Transcription Factor Subunit (FOSB), and palladin, cytoskeletal associated protein (PALLD). No pathways were identified due to the low number of genes regulated by ACM. Higher concentrations of nicotine in the ACM may have stronger effect on gene expression, and is a question for future studies.

There are several strengths in our present study, including that we used cells from four different donors, whilst other publications use 1–2 donors, reducing the possible variabilities between different donors. Another factor of our study is that we used bubbled aerosol/smoke so that we could control for nicotine concentration for side-by-side comparisons of cigarette and ENDS preparations. Furthermore, the findings from these in vitro fully-differentiated primary NHBE cultures have been reported in smokers. For example, current smokers, compared to never smokers, exhibit significantly differentially regulated genes in airway epithelial cells recovered from bronchoscopy associated with oxidative stress (*GPX2* and *ALDH3A1)*, and glutathione and xenobiotic metabolism (*CYP1B1* and *DBDD)* [44]. On the other hand, few clinical studies have investigated the effect of ENDS on gene expression; of those reported, there seems to be fewer gene expression changes as a result of ENDS vaping [20, 45]. One recent investigation observed no significant changes in mRNA or miRNA gene expression in human bronchial epithelial brushings collected from subjects vaping a 50:50 propylene glycol:vegetable glycerin mix for 4 weeks [45]. Another study showed that when cigarette users switched to ENDS, gene expression profiles of the ENDS users were similar to subjects that were former smokers, suggesting that cigarette-induced transcriptional changes revert back to baseline when users switch to ENDS [20]. On the other hand, certain flavoring compounds have been associated with suppressed ciliary beat frequency, mitochondrial respiration, inflammation and immune responses in people that use e-cigarettes [46, 47]. Further investigations into flavoring compounds of ENDS/e-cigarette aerosols is therefore important.

Previously, we have investigated the physiological effects of an acute exposure of WS-CM and ACM on fully-differentiated primary NHBE 3D cultures, including airway mucociliary function, ion channels’ activity, and physiological changes to mucociliary clearance in the airway epithelium [29, 31]. Ion channel function of both ENaC and CFTR was inhibited with cigarette preparations [29]. In this study, WS-CM significantly altered a number of genes associated with regulation of airway mucociliary clearance, including decreased *CFTR* and ENaC genes (*SCNN1B, SCNN1G*), increased mucus production (*MUC5AC, MUC2B*) and regulation of goblet cell differentiation (*SPDEF)*. Altered ion channel function and mucociliary clearance (mucus production) have been strongly linked to lung disease including COPD [48–51]. *FOXA3*, another transcription factor, was also upregulated, and has been shown to be co-localized in goblet cells with SPDEF in mouse lung tissue despite that FOXA3 is not expressed abundantly in the normal lung [50, 52]. In this study, we did not observe any genes associated to mucociliary clearance that were altered with even the highest dose (28 µg/mL Eq-Nic.) of ACM preparations, suggesting that the effects observed after cigarette exposure was not similarly observed with ENDS preparations.

Supporting our findings of in vitro fully-differentiated primary NHBE cultures, several clinical studies have also identified several similar pathways that are regulated by cigarette smoke, including xenobiotic and glutathione metabolism, and inflammatory responses [53–56]. Several canonical pathways were significantly regulated by WS-CM, but not ENDS, in this study. Top pathways affected by WS-CM included upregulation of nicotine degradation and NRF2-mediated oxidative stress response pathways. Oxidative stress is strongly associated with COPD, which in turn can lead to increased risk of lung cancer [53–55]. Activation of oxidative stress and xenobiotic metabolism are included as some of the key characteristic end-points caused by agents known to initiate cancer in humans [56].

Increased expression of metallothionein genes have been associated specifically with acute cigarette smoke exposure, but are suppressed in chronic smokers [57]. Metallothionein, in general, acts as an antioxidant protein. One of the regulators of their expression is Nrf2 signaling pathway (via activation of antioxidant response element), which is a well-known pathway activated by acute cigarette smoke exposure. Nrf2-mediated oxidative stress response pathway was upregulated after 24 h in this study, thus being consistent with metallothionein regulation. A study investigating gene expression in bronchial brushing epithelial cells from individuals who smoked three cigarettes in an acute exposure showed upregulation of several metallothionein genes [57]. In addition, it is known that SPINK1 downregulates metallothionein gene expressions, and therefore metallothionein genes that are involved in the SPINK1 general cancer pathway listed in this study’s IPA canonical pathway analysis. SPINK1 general cancer pathway was significantly inhibited by WS-CM only, with a number of metallothionein genes that were significantly upregulated in this pathway. Metallothionein genes could remain suppressed in former smokers, indicating a persistent risk of lung cancer for these subjects [44], however we did not observe downregulation of metallothionein in this acute exposure study, suggesting that perhaps longer and/or repeated exposures may be needed to induce downregulation. Taken together, the results observed in this study suggest that upregulation of metallothionein was regulated via Nrf2, and not SPINK1 pathway.

Some limitations of this study needs to be considered. Firstly, no reference ENDS product currently exists. Reported results may vary between studies due to different flavorings, base ingredient composition (e.g. ratio of propylene glycol and vegetable glycerin), nicotine concentrations, and preparations (e.g. whole aerosol, media preparations, e-liquid, etc.). A reference cigarette (3R4F) was considered suitable for cigarette smoke comparisons. Secondly, time of treatment significantly affected DEGs, therefore experimental analysis was required to take this time effect into consideration. Primary NHBE cultures are normally kept at ALI, however WS-CM and ACM preparations were kept on the apical surface of the bronchial epithelium for 4 h and 24 h, to allow longer airway exposure. It is possible that leaving fully-differentiated primary NHBE cultures at liquid/liquid interface caused additional stress which may have masked some of the DEGs changed as a result of WS-CM or ACM exposure. Nevertheless, our study shows more differential regulation of gene expression by cigarette smoke than ENDS. Future studies into long-term gene expression changes in in vitro models should be investigated to determine whether changes are prolonged or observed in a more chronic tobacco exposure model.

## Conclusions

We observed a strong differential regulation of gene expression from an acute exposure to cigarette preparations (WS-CM) but not to ENDS preparations (ACM), suggesting a differential response and defense mechanisms towards various tobacco products. Several pathways were regulated by WS-CM including general cancer pathway, oxidative stress, xenobiotic metabolism and mucociliary function. Several gene expression changes related to mucociliary clearance strongly supported physiological changes (decreased CFTR and ENaC ion channels’ activity by WS-CM) we had previously observed in another investigation [29]. The use of fully-differentiated primary NHBE cells for measuring gene expression changes has many advantages since we could use multiple donors (four donors) and achieve a physiological relevant model to study the effects of tobacco products.

## Methods

### Primary human bronchial epithelial cell cultures

Primary NHBE cells were provided by the Nationwide Children’s Hospital Cure Cystic Fibrosis Columbus Epithelial Cell Core, Columbus OH. Cells were isolated from the donor tissues given to the Epithelial Cell Core [30]. Since these cells were provided without identifiers, an exempt status was granted by the Institutional Review Board. The donors were three non-smokers and one with unknown smoking status (see Additional file 2: Table S1 for donor information). Briefly, passage 1 primary NHBE cells were seeded on collagen type IV (0.3 mg/mL; Sigma Aldrich, Saint Louis MO) coated Corning™ Transwells (Fisher Scientific) grown at air–liquid interface (ALI) with PneumaCult™ ALI medium (StemCell Technologies Inc., Tukwila WA) [30]. Medium was changed three times a week and cells were maintained at 37 °C with 5% CO_2_. Cells were used after 4 weeks at ALI once fully differentiated with presence of ciliated cells, as previously published [31].

### Tobacco cigarette and ENDS preparations

Preparation of whole-smoke conditioned-media (WS-CM) from cigarettes and aerosol conditioned media (ACM) from ENDS vapor has been previously described [29, 58, 59]. Nicotine, tobacco specific nitrosamines, and polycyclic aromatic hydrocarbons from WS-CM and ACM preparations were analyzed as previously described [6]. Briefly, WS-CM was prepared by passing smoke from four 3R4F reference cigarettes through Roswell Park Memorial Institute (RPMI) 1640 medium (without phenol red) using the standard ISO method (35 mL puff volume, 60 s puff interval, 2 s puff duration) [59]. ACM was prepared by passing mainstream aerosol (generated using a refillable 10 watts tank device with a 1.5-Ω coil) in an impinger (25 mL Impinger Midget, Ace Glass) containing RPMI media, using a puff profile of 55 mL puff volume, 30 s puff interval, and 5 s puff duration. The tank was filled with tobacco flavor liquid (1.8% nicotine [weight/volume]). Final nicotine content, measured by gas chromatography-flame ionization detection (GC-FID) of the WS-CM and ACM was used for calculating the exposure of cells, expressed as µg/mL of equivalent-nicotine units (Eq-Nic.) units. The final stock concentrations of nicotine in the WS-CM and ACM was 11.1 µg/mL Eq-Nic. and 116.2 µg/mL Eq-Nic., respectively.

### Study design

WS-CM and ACM preparations were diluted in Hank’s Balanced Salt Solution (HBSS) containing calcium and magnesium to various concentrations of Eq-Nic. (Table 2). Exposure concentrations were chosen based on our previously reported investigation of acute ENDS and cigarette effects on cytotoxicity in primary NHBE cultures [29].

**Table 2:** Number of donors used for each treatment dose of WS-CM and ACM at 0 h, 4 h and 24 h. Doses in Eq-Nic. units. Vehicle refers to RPMI medium used to dilute WS-CM and ACM

Exposure time	WS-CM				ACM			
	Vehicle 0 µg/mL	Low 3.6 µg/mL	Medium 7 µg/mL	High 10 µg/mL	Vehicle 0 µg/mL	Low 7 µg/mL	Medium 14 µg/mL	High 28 µg/mL
0 h	4 donors	–	–	–	4 donors	–	–	–
4 h	4 donors	4 donors	4 donors	4 donors	4 donors	4 donors	4 donors	4 donors
24 h	4 donors	4 donors	4 donors	4 donors	4 donors	4 donors	4 donors	4 donors

**Fig. 6:**
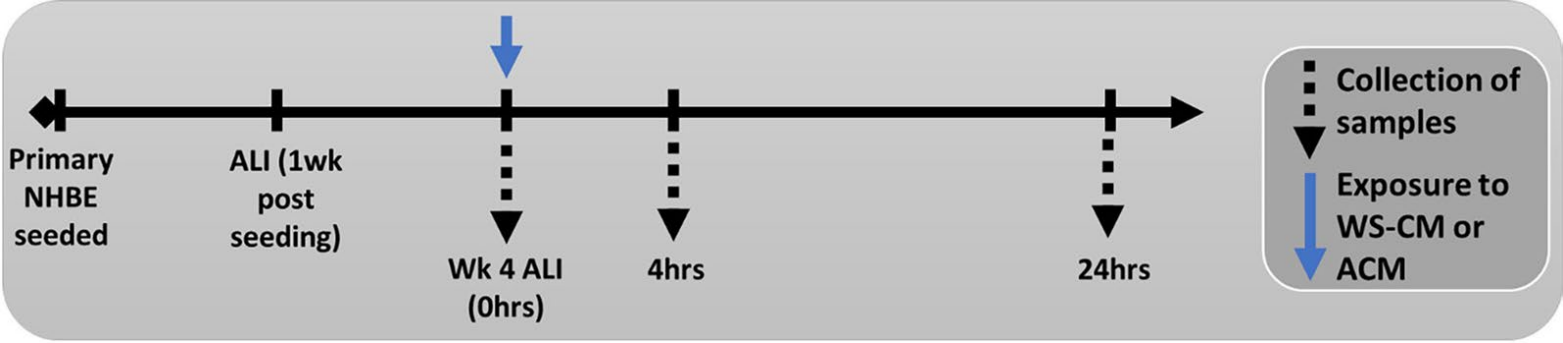
Experimental design used in this study. Fully-differentiated primary NHBE cultures at 4 weeks ALI were treated for 4 h or 24 h with WS-CM or ACM

In order to standardize the different treatments, preparations contained 3.6–28 µg/ml Eq-Nic. units, as previously reported [29]. Control treatments included diluted vehicle (RPMI 1640) in HBSS. All treatments of cells were performed on separate plates to prevent cross-contamination. Primary NHBE cultures were exposed apically to WS-CM or ACM preparations (100 µL volume) for 0 (no treatment), 4 or 24 h (Fig. 6). All exposures were performed in singlets, with four donors.

### Total RNA extraction and purification and sequencing

At treatment endpoints (0, 4 or 24 h), total RNA was isolated using the RNeasy Micro Kit (#74004 Qiagen, Germantown MD). Input RNA quality and quantity was assessed using the Agilent 2100 Bioanalyzer (Agilent Technologies, Santa Clara CA) and Qubit Fluorometer (Thermo Fisher), respectively. Samples with RNA integrity number (RIN) values greater than 7 and RNA concentration greater than 100 ng/µL were sent for sequencing. Sequencing was performed by the Genomics Shared Resources at The Ohio State University, Columbus OH. Messenger RNA (mRNA) sequence libraries were generated with NEBNext® Ultra™ II Directional RNA Library Prep Kit for Illumina (NEB #E7760L) and NEBNext® Poly (A) mRNA Magnetic Isolation Module (NEB #E7490) with an input amount of 200 ng total RNA per sample. Libraries were pooled and sequenced on an Illumina NovaSeq SP flowcell in paired-end 150 bp format (Illumina, San Diego CA) to read yield between 35 and 40 million reads.

### Quality control (QC) and alignment

All RNA-seq datasets (72 samples; in fastq.gz format) were sent to Qiagen for data analysis. Raw data QC was performed for each sample using Array Studio (Qiagen, 2019). Statistics performed included total reads, GC%, N%, adapter%, Q20% and Q30%. Human.B38 was chosen as the genome reference and alignment, and the latest Ensembl.R98 as the gene model. All the sample Fastq files were grouped and aligned using STAR [60] with the default setting. STAR was used in the quantification step to generate the raw count data at the gene level. Normalization was performed using Log Geometric Mean Method [61].

### Principle component analysis (PCA)

PCA was applied to the sample set to identify relationships between different samples and find potential outliers. The first 3 components were used to find potential outliers. PCA was performed based on the normalized count data to detect potential outliers using software Array Studio (Qiagen, 2019). From the PCA, we considered one sample (4 h time-point, vehicle control) as an outlier (Additional file 2: Fig. S1). Therefore, all results described herein were analyzed without the outlier. No particular donor was considered an outlier. None-the-less, any donor variation was accounted for in the downstream statistical model.

### Hierarchical clustering

Hierarchical clustering of the normalized count data was applied to identify grouping of samples based on their gene expression values, using Array Studio (Qiagen, 2019) and Euclidean distance metric as the variable distance method. Samples that were highly correlated were clustered together. The sample level grouping results were further compared to the true metadata to find hidden patterns between samples’ gene expression values and the corresponding metadata.

### Differential expression analysis

DeSeq2 (equivalent to DeSeq V2 of the R package) [61] analysis was performed to identify Differentially Expressed Genes (DEGs) among different comparisons (e.g. WS-CM vs control, ACM vs control, WS-CM 7 µg/mL Eq-Nic vs. ACM 7 µg/mL Eq-Nic.). DEGs were defined as genes whose adjusted p-value was less than 0.05, and log2 fold change greater than 2 or less than − 2. Statistical significance of DEGs was computed using the Benjamini–Hochberg method [62] for multiple comparisons, and adjusted from p-values calculated using the Fisher’s Exact Test. A full statistical model to include all relevant factors (time, treatment and dosage) was built to find out the overall DEGs that are affected by treatments. The full statistical model is described as follows:

$$Model \sim time+treatment+dosage+donor$$

Ingenuity pathway analysis (IPA; QIAGEN Bioinformatics, Redwood City, CA, USA) was used to analyze the differential expression analysis results calculated from comparisons. IPA was also used to investigate differences between different dosages of the same treatment. The statistical significance of the association between genes in the dataset and canonical pathways was measured using the Benjamini–Hochberg method for multiple hypothesis correction [62], and adjusted p-value calculated using Fisher’s Exact Test).

## Supplementary Information

**Additional file 1.** showing the genes listed in Figure 1: “Significant DEGs were identified with low, medium and high dose of WS-CM at 4hrs (Tab 1) and 24hrs (Tab 2) exposure. Genes highlighted yellow were consistently observed in all three doses. Genes highlighted in orange were observed in both medium and high doses, but not low dose. Log2 fold change cut-off is set to ±2 and p-value ≤ 0.05. A list of genes identified across both exposure times (4hrs and 24hrs), and within each dose (low, medium and high) is provided in Tab 3.”

**Additional file 2.** Supplementary Tables 1–5 and Supplementary Figures 1 and 2.

## Data Availability

The datasets used and/or analyzed during the current study available from the corresponding author on reasonable request.

## Abbreviations

ACM: Aerosol conditioned medium
ALI: Air–liquid interface
COPD: Chronic obstructive pulmonary disease
CFTR: Cystic fibrosis transmembrane conductance regulator
ENaC: Sodium epithelial channel
ENDS: Electronic nicotine delivery system
Eq-Nic: Equivalent nicotine units
HPHC: Harmful and potentially harmful constituents
IPA: Ingenuity pathway analysis
LDH: Lactate dehydrogenase
NGS: Next Generation Sequencing
NHBE: Normal human bronchial epithelial
TEER: Trans-epithelial electrical resistance
WS-CM: Whole-smoke conditioned medium
