# Supplementary material for: Differential gene expression of 3D primary human airway cultures exposed to cigarette smoke and electronic nicotine delivery system (ENDS) preparations
Source: BMC Med Genomics. 2022 Apr 3;15:76. doi: 10.1186/s12920-022-01215-x (PMC8978419; doi:10.1186/s12920-022-01215-x)
Supplement: Supplementary file 2 — Additional file 2. Supplementary Tables 1–5 and Supplementary Figures 1 and 2. [file 12920_2022_1215_MOESM2_ESM.docx]

# **Supplementary data**

Sup. Table 1. Donor information (non-identifiable) associated with primary NHBE cells.

| **Donor** | **Sex** | **Age** | **Smoking status** |
| --- | --- | --- | --- |
| A | Female | 19 | Non-smoker |
| B | Female | 54 | Unknown |
| C | Male | 55 | Non-smoker |
| D | Female | 42 | Non-smoker |

**Sup. Figure 1: PCA plots showing the samples’ relationships with each other.** One sample (Donor B) was considered an outlier at 4hrs (orange triangle).


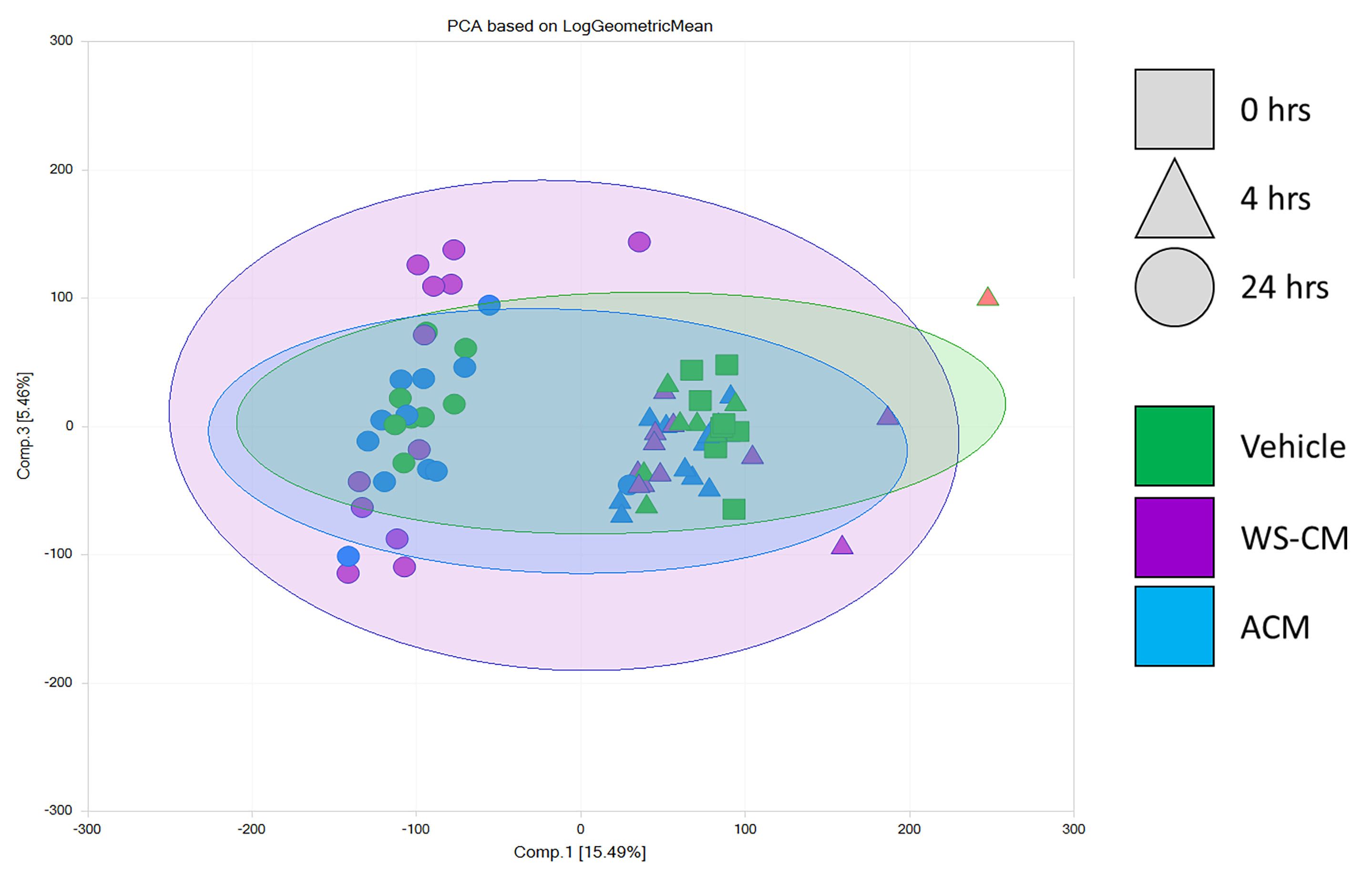


Sup. Table 2. Significant DEGs identified with high dose of ACM (28µg/mL Eq-Nic.) after 4hrs exposure when log2 fold change was lowered to ±1.5 log2 fold change and adjusted p-value ≤ 0.05.

| **Ensembl ID** | **Gene name** | **Estimate** | **Log2 Fold change** | **Raw p-value** | **Adjusted p-value** |
| --- | --- | --- | --- | --- | --- |
| ENSG00000164683 | HEY1 | 0.8475 | 1.7994 | 9.704E-07 | 0.0095 |
| ENSG00000141582 | CBX4 | -0.5058 | -1.4199 | 0.000014569 | 0.0386 |
| ENSG00000169991 | IFF02 | -0.6424 | -1.5609 | 8.6721E-06 | 0.0339 |
| ENSG00000112658 | SRF | -0.7048 | -1.63 | 8.3598E-06 | 0.0473 |
| ENSG00000129116 | PALLD | -0.7281 | -1.6564 | 0.000021778 | 0.0339 |
| ENSG00000118523 | CCN2 | -0.8049 | -1.747 | 8.1319E-06 | 0.0039 |
| ENSG00000125740 | FOSB | -0.9257 | -1.8996 | 1.9233E-07 | 0.0038 |
| ENSG00000143367 | TUFT1 | -0.9582 | -1.9429 | 0.000015814 | 0.0386 |
| ENSG00000283154 | IQCJ-SCHIP1 | -0.9587 | -1.9436 | 0.000010862 | 0.0354 |

**Sup. Figure 2:** **Differential regulation of DEGs by WS-CM when compared to ACM. A)** Venn diagram identifying common DEGs differentially regulated by WS-CM when compared to ACM at 4hrs (146 DEGs) and 24hrs (303 DEGs). **B)** Hierarchical clustering of total DEGs significantly altered under WS-CM (7µg/mL Eq-Nic) conditions compared to ACM (7µg/mL Eq-Nic). Significant DEGs were considered with ±2 log2 fold change and p-value ≤ 0.05, at **B)** 4hrs or **C)**24hrs time-point.


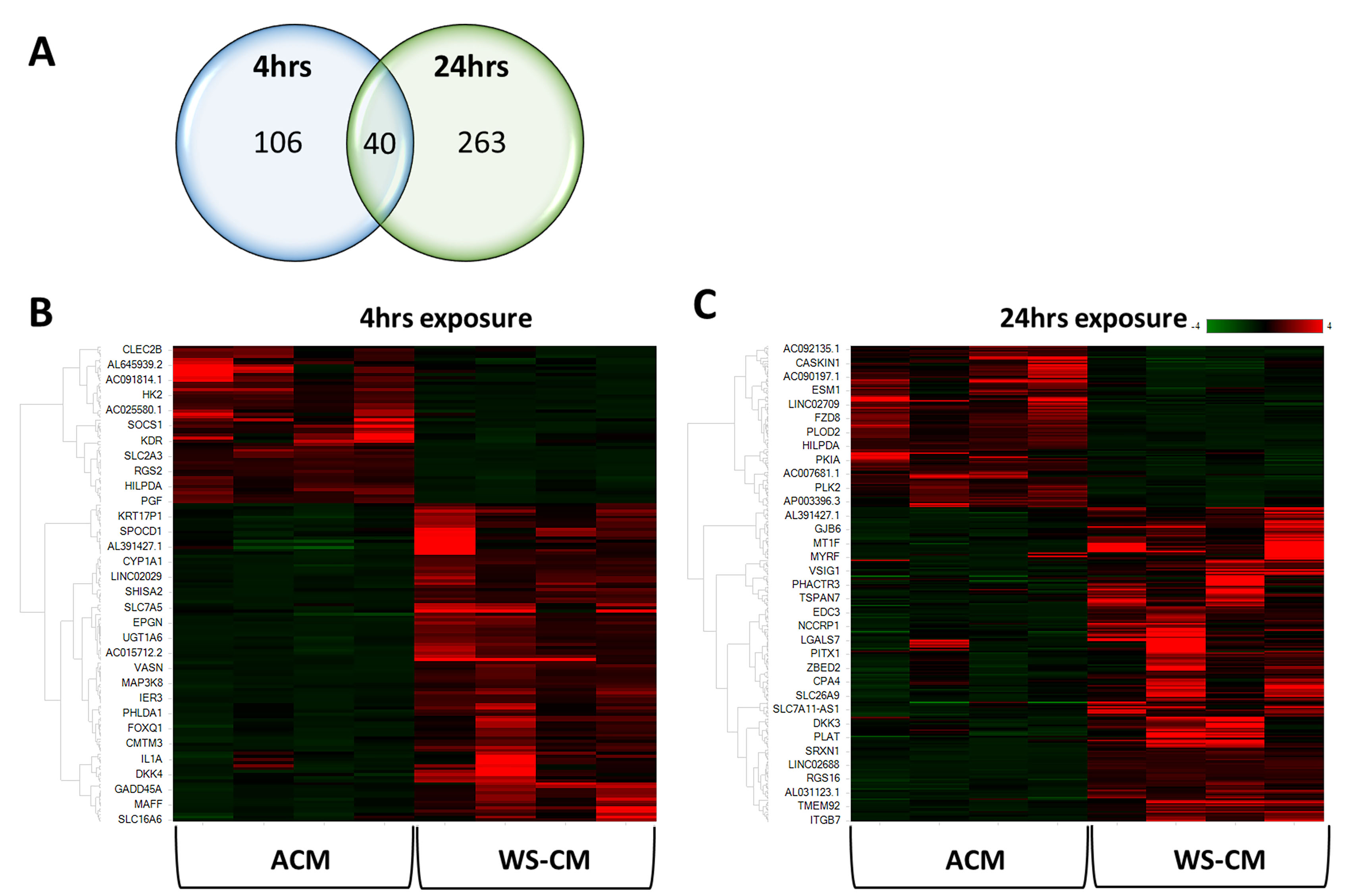


Sup. Table 3. Complete model gene expression differences between WS-CM and ACM, without outliers. We attempted to identify the overall effect of treatment with time effect removed. Fifty nine DEGs were identified as marker genes to differentiate the WS-CM and ACM group (all p-values < 1.0E-5).

| **Ensembl ID** | **Gene Name** | **Estimate** | **Log2 Fold Change** |
| --- | --- | --- | --- |
| ENSG00000196581 | AJAP1 | 1.5754 | 2.9802 |
| ENSG00000000938 | FGR | 1.0554 | 2.0783 |
| ENSG00000117407 | ARTN | 1.1642 | 2.241 |
| ENSG00000173212 | MAB21L3 | -1.0177 | -2.0247 |
| ENSG00000143333 | RGS16 | 1.0121 | 2.0169 |
| ENSG00000073756 | PTGS2 | 1.1317 | 2.1911 |
| ENSG00000143839 | REN | 1.1408 | 2.2051 |
| ENSG00000142224 | IL19 | 1.6223 | 3.0786 |
| ENSG00000162891 | IL20 | 1.3725 | 2.5892 |
| ENSG00000162892 | IL24 | 1.2403 | 2.3624 |
| ENSG00000115758 | ODC1 | 1.392 | 2.6244 |
| ENSG00000138061 | CYP1B1 | 1.8924 | 3.7125 |
| ENSG00000232973 | CYP1B1-AS1 | 1.6767 | 3.197 |
| ENSG00000115602 | IL1RL1 | 1.1934 | 2.2868 |
| ENSG00000121966 | CXCR4 | -1.0318 | -2.0446 |
| ENSG00000244122 | UGT1A7 | 1.0665 | 2.0944 |
| ENSG00000167165 | UGT1A6 | 1.1351 | 2.1963 |
| ENSG00000163870 | TPRA1 | 1.0591 | 2.0836 |
| ENSG00000163659 | TIPARP | 1.5062 | 2.8405 |
| ENSG00000230457 | PA2G4P4 | 1.1161 | 2.1675 |
| ENSG00000137440 | FGFBP1 | 1.0798 | 2.1137 |
| ENSG00000182585 | EPGN | 1.0052 | 2.0072 |
| ENSG00000250033 | SLC7A11-AS1 | 1.2072 | 2.3089 |
| ENSG00000286169 | AHRR | 1.5527 | 2.9337 |
| ENSG00000063438 | AHRR | 1.6431 | 3.1233 |
| ENSG00000145506 | NKD2 | 1.1514 | 2.2213 |
| ENSG00000113083 | LOX | -1.1663 | -2.2443 |
| ENSG00000226281 | AL031123.1 | 1.1816 | 2.2683 |
| ENSG00000124664 | SPDEF | 1.3096 | 2.4786 |
| ENSG00000271820 | AL109910.2 | 1.2308 | 2.3469 |
| ENSG00000135245 | HILPDA | -1.142 | -2.2069 |
| ENSG00000128591 | FLNC | 1.2139 | 2.3196 |
| ENSG00000170379 | TCAF2 | -1.2579 | -2.3914 |
| ENSG00000171388 | APLN | -1.1371 | -2.1993 |
| ENSG00000159167 | STC1 | -1.0761 | -2.1083 |
| ENSG00000104368 | PLAT | 1.0206 | 2.0287 |
| ENSG00000253339 | AC111149.2 | 1.6191 | 3.0719 |
| ENSG00000178919 | FOXE1 | 1.0624 | 2.0884 |
| ENSG00000254872 | LINC02688 | 1.3211 | 2.4986 |
| ENSG00000224251 | AL391427.1 | 1.0042 | 2.0059 |
| ENSG00000177283 | FZD8 | -1.045 | -2.0633 |
| ENSG00000110852 | CLEC2B | -1.1485 | -2.2169 |
| ENSG00000255882 | AC091814.1 | -1.0132 | -2.0184 |
| ENSG00000139626 | ITGB7 | 1.0696 | 2.0988 |
| ENSG00000111432 | FZD10 | 1.3598 | 2.5665 |
| ENSG00000256576 | LINC02361 | 1.0491 | 2.0693 |
| ENSG00000139679 | LPAR6 | 1.4897 | 2.8084 |
| ENSG00000119630 | PGF | -1.0324 | -2.0455 |
| ENSG00000104044 | OCA2 | 1.1897 | 2.2811 |
| ENSG00000178718 | RPP25 | 1.378 | 2.5991 |
| ENSG00000259341 | AC015660.1 | 1.0604 | 2.0855 |
| ENSG00000232386 | AC015712.1 | 1.1472 | 2.2148 |
| ENSG00000184254 | ALDH1A3 | 1.193 | 2.2862 |
| ENSG00000271303 | SRXN1 | 1.0868 | 2.124 |
| ENSG00000178726 | THBD | 1.3295 | 2.5131 |
| ENSG00000134830 | C5AR2 | 1.5297 | 2.8872 |
| ENSG00000187902 | SHISA7 | 1.015 | 2.0208 |
| ENSG00000100292 | HMOX1 | 1.5335 | 2.8948 |
| ENSG00000238120 | LINC01589 | 1.5652 | 2.9591 |

Sup. Table 4. The number of significant differentially expressed genes identified in each Canonical Pathway identified for WS-CM treated cultures after 4hrs. Only Canonical Pathways which are significant (q < 0.05, Z-score > 2) are listed.

|  | **WS-CM vs. vehicle, 4hrs exposure** | | |
| --- | --- | --- | --- |
| **Canonical Pathway** | **Low dose** | **Medium dose** | **High dose** |
| Melatonin Degradation I | 0 | Upregulated – CYP1A1, CYP1B1, UGT1A6, UGT1A7 | Upregulated – CYP1A1, CYP1B1, UGT1A6, UGT1A7 |
| Nicotine Degradation III | 0 | Upregulated – CYP1A1, CYP1B1, UGT1A6, UGT1A7 | Upregulated – CYP1A1, CYP1B1, UGT1A6, UGT1A7 |
| Nicotine Degradation II | 0 | Upregulated – CYP1A1, CYP1B1, UGT1A6, UGT1A7 | Upregulated – CYP1A1, CYP1B1, UGT1A6, UGT1A7 |
| Superpathway of Melatonin Degradation | 0 | Upregulated – CYP1A1, CYP1B1, UGT1A6, UGT1A7 | Upregulated – CYP1A1, CYP1B1, UGT1A6, UGT1A7 |
| Serotonin Degradation | Upregulated – ALDH1A3 | Upregulated – ALDH1A3, UGT1A6, UGT1A7 | Upregulated – ALDH1A3, UGT1A1, UGT1A4, UGT1A6, UGT1A7 |
| Thyroid Hormone Metabolism II (via conjugation and/or degradation) | 0 | Upregulated –UGT1A6, UGT1A7 | Upregulated –UGT1A1, UGT1A4, UGT1A6, UGT1A7 |

Sup. Table 5. The number of significant differentially expressed genes identified in each Canonical Pathway identified for WS-CM treated cultures after 24hrs. Only Canonical Pathways which are significant (q < 0.05, Z-score >2) are listed.

|  | **WS-CM vs. vehicle 24hrs exposure** | | |
| --- | --- | --- | --- |
| **Canonical Pathway** | **Low dose** | **Medium dose** | **High dose** |
| SPINK1 General Cancer Pathway | Upregulated – MT1A, MT1G, MT1M  Downregulated – RAP2B, RASD2 | Upregulated – MT1A, MT1G, MT1M  Downregulated – RAP2B | Upregulated – MT1A, MT1G, MT1M  Downregulated – RAP2B |
| Nicotine Degradation III | Upregulated – CYP1A1, CYP1B1, LARGE2, UGT1A6 | Upregulated – CYP1A1, CYP1B1, LARGE2, UGT1A6 | Upregulated – CYP1A1, CYP1B1, LARGE2, UGT1A6 |
| Melatonin Degradation I | Upregulated – CYP1A1, CYP1B1, LARGE2, UGT1A6 | Upregulated – CYP1A1, CYP1B1, LARGE2, UGT1A6 | Upregulated – CYP1A1, CYP1B1, LARGE2, UGT1A6 |
| NRF2-mediated Oxidative Stress Response | Upregulated – GPX2, HMOX1  Downregulated – MAF, RAP2B | Upregulated – FTL, GCLM, GPX2, HMOX1, MAFG, TXN  Downregulated – MAF, RAP2B | Upregulated – FTL, GCLM, GPX2, HMOX1, MAFG, TXN  Downregulated – MAF, RAP2B |
| Gαs Signaling | Upregulated – ADCY9, ADRB1, GNG4, RAPGEF3, RYR1, VIPR1  Downregulated – HCAR1 | Upregulated – ADCY1, ADCY9, ADRB1, GNG4, GNG7, GPER1, RAPGEF3, RYR1, VIPR1  Downregulated – HCAR1 | Upregulated – ADCY9, GPER1, RAPGEF3, RYR1  Downregulated – HCAR1 |
| Gluconeogenesis I | Downregulated – ENO2, GAPDH, GPI, PGAM1, PGK1 | Downregulated – ENO2, GAPDH, GPI, PGAM1 | Downregulated – ENO2, GAPDH, GPI |
| Glycolysis I | Downregulated – ENO2, GAPDH, GPI, PGAM1, PGK1 | Downregulated – ENO2, GAPDH, GPI, PGAM1 | Downregulated – ENO2, GAPDH, GPI |

# **Additional file 1**

Significant DEGs were identified with low, medium and high dose of WS-CM at 4hrs (Tab 1) and 24hrs (Tab 2) exposure. Genes highlighted yellow were consistently observed in all three doses. Genes highlighted in orange were observed in both medium and high doses, but not low dose. Log2 fold change cut-off is set to ±2 and p-value ≤ 0.05. A list of genes identified across both exposure times (4hrs and 24hrs), and within each dose (low, medium and high) is provided in Tab 3.
